# Changes in Preventive Behavior During the First 3 Months of the COVID-19 Outbreak in Iran

**DOI:** 10.1017/dmp.2020.378

**Published:** 2020-10-12

**Authors:** Abbas Shamsalinia, Sepideh Mohammadi, Fatemeh Ghaffari, Tajmohammad Arazi

**Affiliations:** Department of Nursing, Nursing Care Research Center, Health Research Institute, Babol University of Medical Sciences, Babol, I.R. Iran; Department of Nursing, Nursing Care Research Center, Health Research Institute, Babol University of Medical Sciences, Babol, I.R. Iran; Department of Nursing and Operating Room, Neyshabur University of Medical Sciences, Neyshabur, I.R. Iran

**Keywords:** COVID-19, novel coronavirus, preventive behavior, protective behaviors

## Abstract

**Objectives:**

Iran is facing a big challenge controlling the coronavirus disease 2019 (COVID-19) outbreak, and it is unclear to how individuals are engaging in preventive behaviors. This study aimed to investigate changes in preventive behaviors during the first 3 mo of the COVID-19 outbreak in Iran.

**Method:**

This cross-sectional survey was conducted on 1477 adults aged 18 y and older in 31 provinces of Iran. Data were collected by an anonymous online questionnaire.

**Result:**

Overall, engagement in preventive behaviors was relatively acceptable, and more than 45% of subjects always carried out all preventive behaviors. Engaging in all preventive behaviors had a peak in the second month and obviously declined during third month. Engagement in some preventive behaviors, such as “wearing a face mask” and “keeping a safe distance from others,” was observed less than other behaviors. There was a statistically significant difference in the engagement in preventive behaviors by gender and occupation (*P* < 0.001).

**Conclusions:**

Although engagement in preventive behaviors was relatively acceptable for the first 2 mo of the outbreak, it has declined gradually. This is a warning for public health decision makers. COVID-19 is still a crucial issue in Iran, and it is necessary that government decision be based on the fact that Iranian people must live with a coronavirus for months, with full caution and compliance toward all preventive care protocols.

In December 2019, an outbreak of atypical pneumonia was discovered in Wuhan City, Hubei Province, China.^1^ A novel coronavirus disease 2019 (COVID-19) was identified as the cause of atypical pneumonia outbreak in Wuhan and has spread quickly around the world.^2^ First confirmed cases of COVID-19 were reported on February 19, 2020 in Iran and at the end of February, the virus had spread to most provinces of the country.^3^ On June 6, 2020, the Ministry of Health and Medical Education (MOHME) overall reported 167,156 coronavirus cases, 8134 deaths, and 129741 recovered cases.^4^ Iran has struggled to control the outbreak since it announced the country’s first coronavirus cases nearly 4 mo ago at the time of this writing, and the incidence rate of coronavirus is still high among Iranian people.^5^

Like other countries, a special focus of the Iranian government has been reducing transmission of the disease to flatten the peak of outbreak.^6^ Achieving this goal can be difficult in the case of novel coronavirus due to its power of transmissibility and severity, asymptomatic carriers of novel coronavirus, lack of specific drugs or vaccines, and high mortality rate particularly among older adults with chronic disease.^7,8^ However, the success of these measures rely mostly on quickly changing human behaviors, which are based on populations’ ability to understand risks associated with the novel coronavirus and adjust their behaviors accordingly.^9^ Protective behaviors are the main options to prevent transmission of the virus as vaccination or specific treatments are not available.^10^ Protective behaviors recommended in response to outbreak of novel Coronavirus include mask wearing, staying at home, quarantine restrictions, keeping social distance from others, washing hands for at least 20 s, cleaning surfaces, covering the mouth and nose with a tissue when coughing or sneezing, and avoiding crowds and public transportation.^11^

Novel coronavirus is continuing its spread across the world, and there is no evidence right now as to when it will really be over.^2^ Prolonged quarantine, isolation, uncertainty over health, jobs, finances, and listening to negative news are usually accompanied by unpleasant side effects, including anxiety, stress, fear, hopeless, depression, and posttraumatic stress.^12,13^ Although low level stressors help individuals to prepare to face challenges and behave according to what the situation demands, usually long-term stressors can lead to emotional exhaustion and burnout. Emotional exhaustion can affect behavior and may lead to apathy.^14^ According to previous studies, perceived risk and experienced stress affect people’s behavior during a pandemic.^15-17^ In the face of pandemic novel coronavirus, it is crucial to figure out how populations behave to reduce the risk of virus transmission. Public preventive behaviors are expected to change over the period of the novel coronavirus outbreak because government policies, information about disease and psychological situation of people change as the virus pandemic develops. However, few studies pay attention to the dynamic changes of individuals’ preventive behavior over the novel coronavirus outbreak. It is unknown how preventive behaviors in Iranian people have changed during first 3 mo of outbreak. This study aimed to investigate Iranian preventive behaviors separately for each month after the first confirmed cases of COVID-19 in Iran and understand the dynamic changes of behaviors over this period.

## METHODS

### Design and Sampling

A cross-sectional survey of 1477 adults aged 18 y and older in 31 provinces of Iran was conducted to assess individuals’ preventive behaviors during the first 3 mo of the outbreak. Data were collected by an anonymous online questionnaire and was compiled over 7 days from May 21, 2020, to May 28, 2020. Inclusion criteria were: ages above 18 y, ability to read Persian, and access to social networks. After data collection, incomplete questionnaires were excluded. A snowball and convenience sampling were adopted. The major platforms for distribution of the online questionnaire were social networks, including WhatsApp, Telegram, and Instagram. These applications are the most popular social media sites in Iran. First, the online questionnaire was disseminated to news, educational, entertainment, and university students’ groups in social media apps and then members were encouraged to pass it on to their friends and other groups. To prevent duplicate responses to our online questionnaire, we chose Internet provider (IP)-based duplicate protection.

### Data Collection Tool

Data were collected with an anonymous online questionnaire, including 2 parts; Part 1 was related to demographic characteristics data were collected on age, gender, province, level of education, employment status, and history of the infection with COVID-19 (Table 1).

**TABLE 1:** Baseline Characteristics of Study Sample (*N* = 1477)

Variable		N (%)
Gender	Female	857 (58.03)
	Male	620 (41.97)
Age (year)	18-29	366 (24.77)
	30-39	457 (30.94)
	40-49	297 (20.1)
	50-59	222 (15.03)
	>60	135 (9.14)
Occupation	Enterprise employees/institution workers	473 (32.02)
	Self-employment	259 (17.53)
	Health workers	261 (17.67)
	University student	217 (14.69)
	Housekeeper	188 (12.72)
	Others	79 (5.34)
Education level	Under diploma	160 (10.83)
	Diploma and high diploma	354 (23.96)
	Bachelor	512 (34.66)
	Master	277 (18.75)
	Higher	174 (11.78)
Infected with the COVID-19	No	1385 (93.77)
	Yes	92 (6.23)

Part 2 was the preventive behaviors against COVID-19 questionnaire (PBCQ). A 3-point Likert scale, “never,” “sometimes,” and “always” was developed to evaluate individuals’ preventive behaviors against COVID-19. The PBCQ was a self-administered questionnaire and consisted of 12 items, including wearing a face mask in public places; staying at home; avoiding public places as much as possible; keeping social distance; washing hands for at least 20 s; washing or disinfecting bought items; covering the mouth and nose with a tissue when coughing or sneezing; discarding gloves, mask, tissues, and all infected things into closed bag; avoiding shaking hands, hugging, or kissing people; avoiding use of public transport; avoiding reusing disposable gloves and masks; and avoiding going to parties or relative’s/friends’ houses. To assess individuals’ protective behaviors against COVID-19 in each month separately, all the PBCQ items were rated never to always separately for 3 mo. The first cases of novel coronavirus were officially announced in Iran on February 19, 2020. So, we assessed individuals’ preventive behaviors against COVID-19 from February 19 to May 20: “February 19 to March 19 (first month)”, “March 20 to April 19 (second month)”, and “April 31 to May 20 (third month).”

Content validity was used to determine the validity of the PBCQ. We asked 10 infectious disease doctors and health faculty members who had expertise in novel coronavirus to revise and validate PBCQ, and all of their recommendations were implemented. Finally, content validity index was calculated for the PBCQ and all items were higher than 0.8. The internal consistency method was used to ensure the reliability of the PBC scale. We asked 30 individuals to fill out the PBCQ, and then we calculated Cronbach’s alpha which was equal to 0.89.

### Ethical Approval

The medical ethics committee of Babol University of Medical Sciences approved the study (approval number: IR.MUBABOL.REC.1399.152). An electronic informed consent was obtained from research subjects before collecting data and they were assured of confidentiality and anonymity.

## RESULTS

### Preventive Behaviors

**FIGURE 1:**
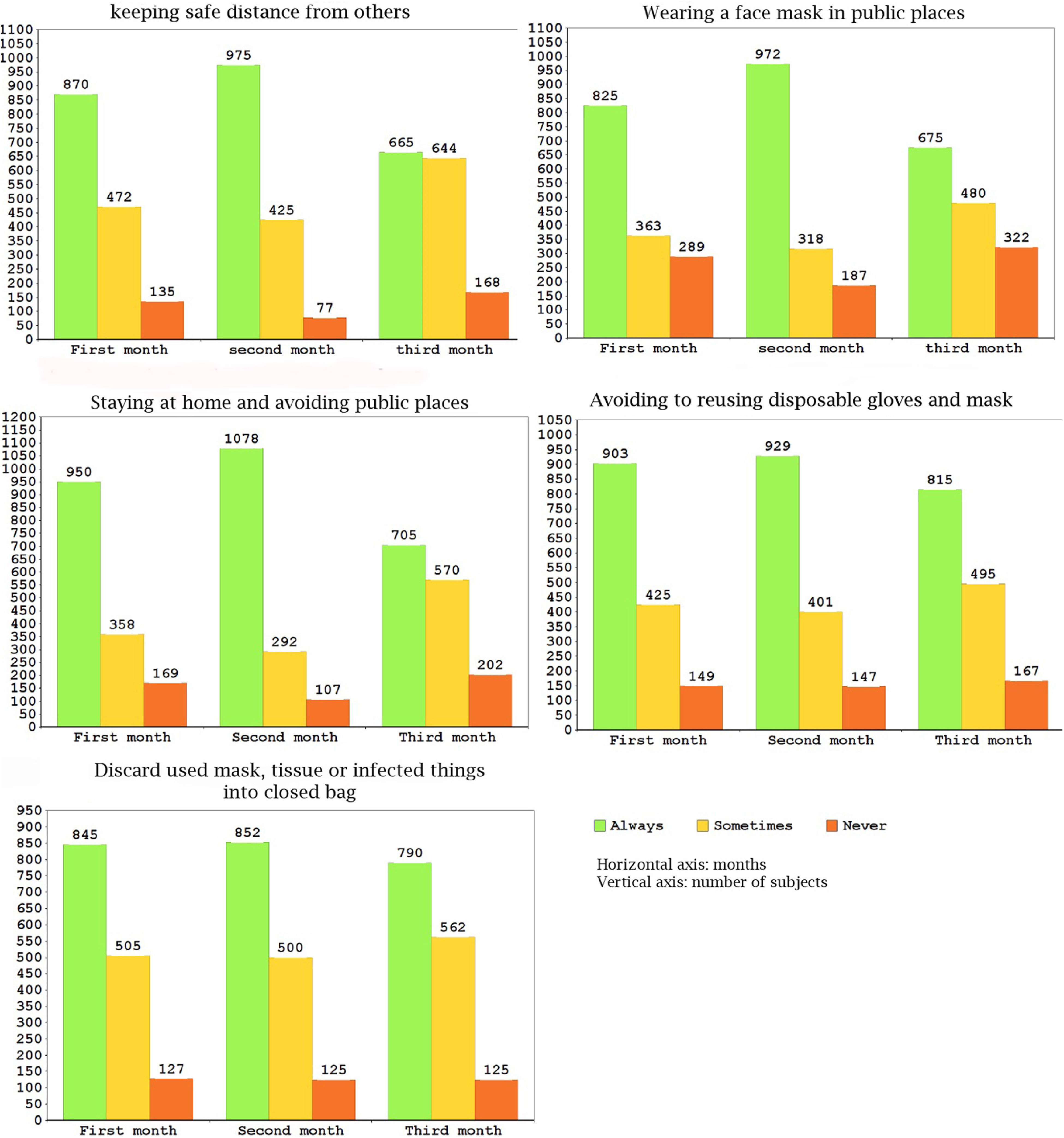
Distribution of Responses to the 5 Items with the Lowest Level of Reported Engagement in Preventive Behavior.

**FIGURE 2:**
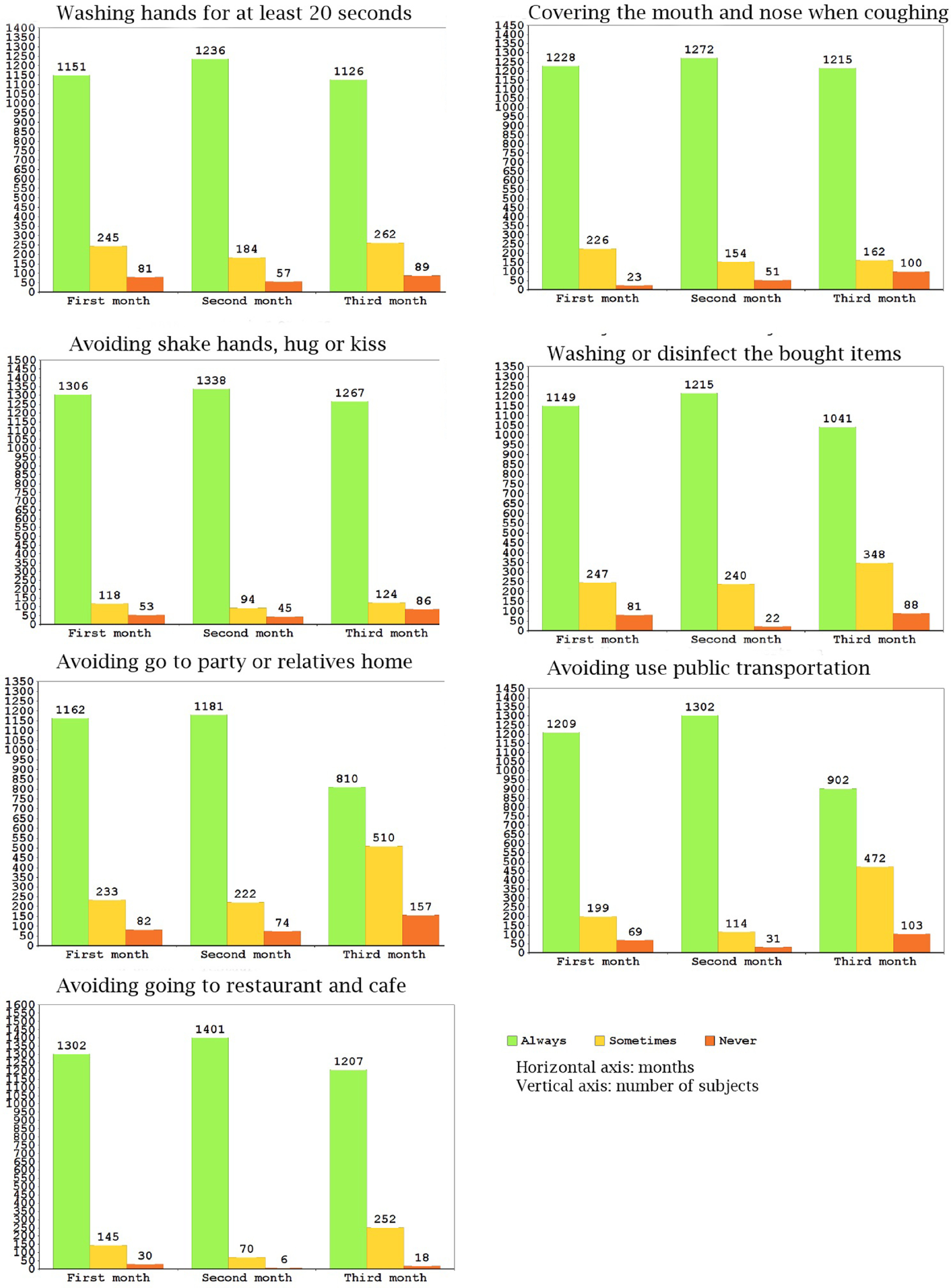
Distribution of Responses to the 7 Items with the highest Level of Reported Engagement in Preventive Behavior.

Our analyses indicate that engagement in all preventive behaviors had a peak in the second month (March 20 to April 19). Engaging in the most of preventive behaviors obviously declined during the third month (April 31 to May 20), such as “wearing a face mask in public places,” “staying at home and avoiding public places as much as possible,” “keeping a safe distance from others,” “washing or disinfecting the bought items,” “avoiding use of public transport,” and “avoiding going to party or relatives/friends’ houses” (Figures 1 and 2).

The percentage of respondents’ engagement in preventive behaviors was calculated by 3 mo separately and overall. Overall, engagement in preventive behaviors was relatively acceptable and more than 45% of participants always carried out all preventive behaviors (Table 2). Engagement in some preventive behaviors, such as wearing a face mask in public places; keeping a safe distance from others; avoiding reusing disposable gloves and mask and discarding gloves, masks, and infected things into a closed bag; was observed lees than other behaviors. The highest level of engagement was related to some behaviors such as avoiding shaking hands, hugging, or kissing; avoiding going to restaurant and café; and covering the mouth and nose when coughing (Figures 1 and 2).

**TABLE 2:** Percentage of Responses to Items Regarding Preventive Behaviors Over Time

	1st mo			2nd mo			3rd mo			Entire 3 mo		
	Always	Sometimes	Never	Always	Sometimes	Never	Always	Sometimes	Never	Always	Sometimes	Never
Keeping a safe distance from others	58.9%	32%	9.1%	66%	28.8%	5.2%	45%	43.6%	11.4%	56.6%	34.8%	8.6%
Wearing a face mask in public places	55.9%	24.5%	19.6%	65.8%	21.5%	12.7%	45.7%	32.5%	21.8%	55.8%	26.2%	18%
Washing hands for at least 20 s	77.9%	16.6%	5.5%	83.8%	12.4%	3.9%	76.2%	17.7%	6.0%	79.3%	15.6%	5.1%
Staying at home and avoiding public places	64.3%	24.2%	11.5%	73%	19.8%	7.2%	47.7%	38.6%	13.7%	61.7%	27.5%	10.8%
Covering the mouth and nose when coughing	83.2%	15.2%	1.6%	86.1%	10.4%	3.5%	82.2%	11%	6.8%	83.8%	12.2%	3.9%
Avoiding shake hands, hugging, or kissing	88.4%	8%	3.6%	90.6%	6.4%	3%	85.8%	8.4%	5.8%	88.3%	7.6%	4.2%
Washing or disinfect the bought items	79.8%	14.7%	5.5%	82.4%	16.1%	1.5%	70.5%	23.5%	6%	77.6%	18.1%	4.3%
Avoiding go to party or relatives home	78.7%	15.8%	5.6%	80%	15%	5%	55%	34.4%	10.6%	71.2%	21.7%	7.1%
Avoiding to reusing disposable gloves and mask	61.3%	28.6%	10.1%	63%	27%	10%	55.3%	33.4%	11.3%	59.9%	29.7%	10.4%
Discard used mask, tissue, or infected things into closed bag	57.8%	33.7%	8.5%	53.6%	37.9%	8.5%	81.9%	13.5%	4.7%	56.3%	35.2%	8.5%
Avoiding use public transportation	81.9%	13.5%	4.6%	88.2%	9.7%	2.1%	67.6%	25.5%	6.9%	79.2%	16.2%	4.6%
Avoiding going to restaurant and cafe	88%	10%	2%	94.7%	4.8%	0.5%	81.8%	17%	1.2%	88.2%	10.6%	1.2%

The subjects reported engaging in the 3 most important protective behaviors stratified by demographic variables. There was no statistically significant difference in the engaging in preventive behaviors by age, education level, and infection with COVID-19 (*P* > 0.05). There were statistically significant differences in the engagement in preventive behaviors by gender and occupation, as shown in Table 3. Engagement in the preventive behaviors was significantly higher in females and health workers than other participants (*P* < 0.001).

**TABLE 3:** Percentage of Engagement in the Most Important Preventive Behaviors in Iranian Population Stratified by Gender and Occupation

		Keeping a Safe Distance From Others					Wearing a Face Mask in Public Places					Washing Hands for at Least 20 s				
		Always	Some-times	Never	Analysis		Always	Some-times	Never	Analysis		Always	Some-times	Never	Analysis	
					*P*	χ^2^				*P*	χ^2^				*P*	χ^2^
Gender	Female	71.4%	23.1%	5.5%	0.00	174.4	70.5%	16.5%	13.1%	0.00	205.0	80%	17.2%	2.8%	0.00	24.2
	Male	36.9%	51.5%	11.6%			36%	49.2%	14.8%			78.4%	13.4%	8.2%		
Occupation	Enterprise employees	54.5%	37%	8.5%	0.00	126.4	52.9%	33.4%	13.7%	0.00	112.04	79.9%	14.4%	5.7%	0.00	110.5
	Self-employment	51.5%	41.7%	5.8%			52.5%	30.1%	17.4%			75.7%	20.8%	3.5%		
	Health worker	80.5%	18%	1.5%			79.3%	20.3%	0.4%			98.5%	1.1%	0.4%		
	University student	52.1%	33.2%	14.7%			51.6%	36.3%	22.1%			70%	20.3%	9.7%		
	House keeper	51.1%	43.6%	5.3%			43.1%	43.6%	13.3%			73.4%	23.9%	2.7%		
	Other	53.2%	24.1%	22.8%			51.9%	22.8%	25.3%			64.6%	20.3%	15.2%		

## DISCUSSION

The noticeable global impact of COVID-19 not only has a strong effect on people who were infected with the coronavirus, but also extends to all people in the affected countries. Iran, being among the first countries after China to deal with the COVID-19 outbreak, and almost 4 mo after the government confirmed the first known case (February 19, 2020), the incidence of the disease in some cities of Iran remains high.^6^ The (Iranian) government has expressed serious concern about the new waves of the outbreak, during October, November, and December 2020. Given the ongoing rampant nature of COVID-19 and in the absence of special pharmaceutical interventions, changing behavior is the main key to preventing transmission of the virus.^3^ Understanding the public response and preventive actions to break the transmission chain of COVID-19 during first 3 mo after confirmation COVID-19 in Iran, may help government and health ministry to improve their implication about new waves of the outbreak and new infectious diseases in the future.

Based on the results of this study, engagement in preventive behaviors in the second month of outbreak (March 20 to April 19) considered better than first and third months. Nowruz is the name of the Iranian New Year and occurs on March 21. For 13 days, Iranians celebrate with various traditions, and most importantly, with visiting family and traveling around Iran. Iranian authorities stated that the most important way to control the outbreak on Nowruz is social distancing by reducing traveling, and canceling unnecessary trips, as well as staying at home under voluntary home quarantine.^18,19^ The health ministry announced that public screening to detect coronavirus would be underway at airports, railway stations, and the city entrances and exits during Nowruz. Also, restrictions have been put in place for all travelers arriving from outside the province and all travel deemed unnecessary has been prohibited.^19^ Imposing severe restrictions on traveling during Nowruz is a justifiable reason for improving the respondents’ compliance toward some preventive behaviors such as “staying at home” in the second month of the outbreak. Moreover, improving the respondents’ compliance toward preventive behaviors coincided with a first peak of COVID-19 deaths and new cases in Iran.

According to a report released by the Iranian Health Ministry, the COVID-19 epidemic trend in Iran raised from March 22, 2020, and reached a peak during March 24, 2020, to April 8, 2020. The highest number of coronavirus deaths was recorded during March 15, 2020 to April 15, 2020.^4^ A study carried out in the United Kingdom found that fear of COVID-19 and its morbidity or mortality rates was an important predictor of positive behavior change.^20^ In the other studies, an association has been found between the perceived severity of disease during an outbreak and carrying out preventive behaviors.^21,22^ In addition to the high morbidity and mortality rate over this period, the government imposed the most restrictions on people traveling, quarantine in cities, and closing public place during this time. Government pressure also seems to have been effective in improving preventive behaviors during second month. Social and governments pressure has been shown to be related to wearing face masks during the outbreak of severe acute respiratory syndrome (SARS)^23^ and in term of other preventive behaviors, such as personal cleanliness and home hygiene among adults.^24^ A relationship has been found in Canada between social pressure and compliance with quarantine.^25^

Engagement in most of preventive behaviors obviously declined during third month. Engaging in the preventive behaviors is expected to change over the period of the novel coronavirus outbreak because government policies, information about disease, and psychological situation of people change as the virus pandemic develops.^16^ Researches on past outbreaks have shown that negative emotions, such as fear of disease and anxiety, motivate a range of preventive behaviors that reduce the engagement in precarious behaviors. It should be noted that, gradually people cope with fear of new virus and they deal with feelings of anxiety; therefore, a reduction in preventive behavior due to coping of people with fear is predictable. Other studies have shown that anxiety, stress, and fear of infection in the time of pandemic have a functional role, and are related to increased compliance of preventive behaviors. However, long-term stress and fear of corona virus was related to decreased psychological, physical, and social well-being.^20,26^ In terms of the psychological impact of COVID-19 outbreak in Iran, a study showed that the severity of psychological distress, such as stress, depression, and mental exhaustion, was high among the Iranian population.^27^ It seems that, emotional exhaustion during first 2 mo of coronavirus isolation and easing restrictions during third month played an important role in reducing compliance toward preventive behaviors.

Our analyses indicate that overall compliance toward preventive behaviors was relatively acceptable, and more than 45% of participants always carried out all preventive behaviors. A study carried out in the United States characterized people’s engagement in preventive behaviors over the first week of the COVID-19 pandemic, and the results indicated that most of the subjects reported carrying out the protective behaviors with increasing frequency.^16^ A study carried out in Myanmar found that Myanmar adults have low knowledge and inadequate preventive behaviors to prevent spread of the novel coronavirus. According to their study, only 22% reported good preventive behaviors, 45% of participants frequently wash their hands, 47% of participants always cover the mouth and nose during sneezing or coughing, and just 34% of participants avoided travel. This study indicated that the differences in carrying out protective behaviors between Myanmar and other countries might be due to the lack of knowledge toward the novel coronavirus in this country.^28^

Our findings indicate that wearing a face mask in public places and avoiding reusing disposable gloves and masks were in the lowest levels of compliance. It may be due to shortage of face masks and gloves and rising cost of face masks in Iran. In addition to this problem, contradicting recommendations were provided by health authorities on the efficacy of face masks to limit the transmission of COVID-19.^29^ It has been found that there is association between perceived efficacy of behavior and carrying out preventive behaviors during the outbreak of swine flu.^30^ Studies have found a relationship between perceived efficacy of wearing face masks to protect against influenza and SARS and intentions to carrying out this behavior.^31-33^ An association also has been found between the perceived barriers and costs to protective behaviors and carrying them out. A qualitative study carried out in the United Kingdom found that people experienced some barriers to carrying out protective behaviors in the event of a pandemic, such as shortages of sanitary products or lack of space to keep social distance from other people.^6^

Our findings demonstrated that engagement in the preventive behaviors was significantly higher in females and health workers than other participants. Our results were similar to another study during COVID-19.^34^ According to some studies, during a pandemic, compliance rates were associated with some demographic characteristics: females and health-care workers had higher overall compliance scores than males and other occupational groups.^34,35^ Compared with the general population, health-care providers have higher health literacy as well as, due to duty calls, they are more likely to come in contact with COVID-19 carriers, putting them at a greater risk of contracting the infection and spreading it to others.^36^ Therefore, it is to be expected that engagement in the preventive behaviors was significantly higher in the health-care providers than other occupational groups. Moreover, psychological studies showed that the level of COVID-19-related anxiety was higher among women and health-care providers. Studies showed that disease-related anxiety is a powerful motivating factor that increases the engagement in preventive behaviors.^12,36^

## CONCLUSIONS

The importance of this study lies in providing insights into public response and preventive actions during a critical phase in the developing situation. Our findings indicated that, although public compliance toward preventive behaviors was relatively acceptable during the first 2 mo, it has declined gradually. This is a warning for public health decision makers. COVID-19 is still a crucial issue in Iran, and it is necessary that government decisions be based on the fact that Iranian people must live with the coronavirus for months, with full caution and compliance toward all preventive care protocols. The government should focus on reducing the anxiety and stress associated with COVID-19, managing emotional exhaustion, increasing the risk awareness and encouraging appropriate behavioral change. Also, it is important that the government effectively explain the preventive measures to all citizens if it is to improve public response.
